# S100A8/A9 innate immune signaling as a distinct mechanism driving progression of smoking-related breast cancers

**DOI:** 10.1038/s41388-025-03276-5

**Published:** 2025-01-25

**Authors:** Samson Mugisha, Shahnawaz A. Baba, Shreyas Labhsetwar, Devam Dave, Aran Zakeri, Richard Klemke, Jay S. Desgrosellier

**Affiliations:** 1 Department of Pathology, University of California, San Diego, La Jolla, USA; 2 Moores Cancer Center, University of California, San Diego, La Jolla, USA

**Keywords:** Breast cancer, Tumour heterogeneity, Cytokines

## Abstract

Smoking plays an underappreciated role in breast cancer progression, increasing recurrence and mortality in patients. Here, we show that S100A8/A9 innate immune signaling is a molecular mechanism that identifies smoking-related breast cancers and underlies their enhanced malignancy. In contrast to acute exposure, chronic nicotine increased tumorigenicity and reprogrammed breast cancer cells to express innate immune response genes. This required the α7 nicotinic acetylcholine receptor, which elicited dynamic changes in cell differentiation, proliferation, and expression of secreted cytokines, such as S100A8 and S100A9, as assessed by unbiased scRNA-seq. Indeed, pharmacologic or genetic inhibition of S100A8/A9-RAGE receptor signaling blocked nicotine’s tumor-promoting effects. We also discovered Syntaphilin (*SNPH*) as an S100A8/A9-dependent gene enriched specifically in estrogen receptor-negative (ER^-^) cancers from former smokers, linking this response to patient disease. Together, our findings describe a new α7 nAChR-S100A8/A9-Syntaphilin immune signaling module that drives nicotine-induced tumor progression and distinguishes smoking-related patient disease as a distinct subset of aggressive breast cancers.

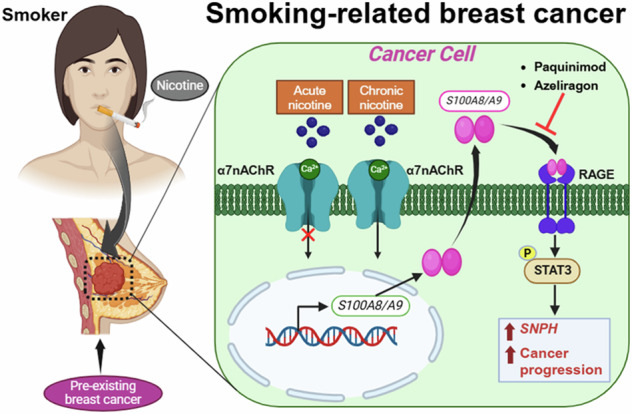

## Introduction

Tobacco-use is associated with an increased risk of breast cancer progression [1–3]. While smoking can enhance the possibility of developing new breast cancers [4–6], strong clinical evidence also links tobacco smoke with a significantly worse outcome in patients with pre-existing disease [1–3]. These effects were not limited to current smokers, as former smokers with high lifetime exposure ( > 20 pack-years) had a 54% increased risk of breast cancer-specific mortality [1]. Thus, smoking cessation does not remove the likelihood of disease progression for some patients with high exposure. Notably, these effects were dose-dependent [2], since former smokers with >35 pack-years displayed a further increased probability of recurrence and mortality. They were also independent of subtype, as effects occurred in both ER^+^ and ER^-^ cancers [1, 2]. In contrast, former smokers with low exposure have a similar risk as those that never smoked [1, 2]. These robust findings include data from nearly 10,000 patients in three clinical studies [1, 2]. Importantly, former smokers with >20 pack-years represented 21.2% of patients [2], and thus constitute a significant fraction of breast cancers. Together, these prior studies highlight the importance of lifetime exposure to tobacco products, not just smoking status, in determining the risk of poor outcome for a large segment of breast cancer patients.

Despite discovery of a clinical link between breast cancer progression and high lifetime exposure to tobacco smoke over a decade ago, the cause is still largely unknown. In fact, the degree to which smoking alters breast cancer cells directly remains a relatively unexplored question. In lung cancer, studies showed that nicotine, the addictive component of cigarette smoke, may be important for the progression of pre-existing disease [7]. However, studies of nicotine in breast cancer cells have yielded only modest and transient effects on EMT markers and cell proliferation [8–11], leading to speculation that its primary impact may be on the tumor microenvironment [12–14]. While some studies suggest a potential direct effect of nicotine on breast cancer cells through its receptors [8, 11, 15], others indicate an indirect or systemic role [13, 15]. Since nicotine is non-carcinogenic, this indicates that it likely affects tumor progression by enhancing particular cell types or signaling states, rather than inducing oncogenic transformation. Thus, how nicotine affects cell signaling and gene expression programs in breast cancer cells is a critical unresolved question that may help explain differences in outcome due to lifetime tobacco-use.

In the present study we sought to address these gaps in the field by investigating a potential direct impact of chronic nicotine on breast cancer cells in comparison to acute treatment. Unlike lung or head and neck cancers, there is no direct contact between breast tissue and tobacco smoke. However, nicotine is readily absorbed into the bloodstream and thus able to directly impact perfused breast cancer cells. Furthermore, previous studies have already shown expression of nicotine receptors in breast cancer cell lines [16], indicating their ability to directly respond to nicotine treatment. While smoking may increase the overall risk of developing breast cancer [4–6], the studies described here focus on how it impacts pre-existing disease, resulting in increased recurrence and mortality in patients [1, 2]. Our in-depth and unbiased approach yielded surprising differences in innate immune gene expression reprogramming due to chronic nicotine, providing a potential molecular basis for the enhanced progression of smoking-related breast cancers.

## Results

### Chronic nicotine drives tumor-initiating cell properties

**Fig. 1:**
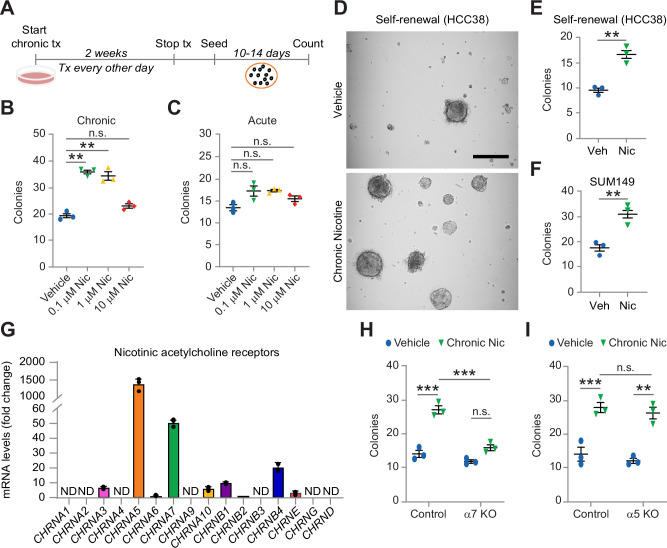
Chronic, not acute, nicotine enhances tumorspheres via α7 nAChR. **A** Schematic outlining the experimental design for HCC38 cells treated with chronic nicotine and seeded into methylcellulose tumorsphere assays. **B**, **C** Colony counts comparing cell treated with vehicle (Veh) and chronic **B** or acute **C** nicotine (Nic) at the indicated doses. Statistics by one-way ANOVA and Tukey’s multiple comparisons test. **D**, **E** Representative images **D** and colony counts **E** of secondary tumorspheres from self-renewal assays with HCC38 cells chronically treated with vehicle or 100 nM nicotine. (**D**) Scale bar, 200 μm. **F** Primary tumorsphere assays with SUM149 cells chronically treated with vehicle or 100 nM nicotine and seeded into methylcellulose. **E**, **F** Statistics by two-tailed Student’s t-test. **G** QPCR for select nAChR genes in parental HCC38 cells. Samples were run in duplicate with GAPDH as a loading control. Expression is shown relative to *CHRNB2*. ND = not determined using a CT value threshold of 35. **H**, **I** Methylcellulose tumorsphere assays with control or KO of the α7 or α5 nAChR in HCC38 cells followed by chronic treatment with vehicle or 100 nM nicotine. Statistics by two-way ANOVA and Tukey’s multiple comparisons test. **B**, **C** and **E**–**I** Data represent the mean ± s.e.m. **P < 0.01 and ***P < 0.001. n.s. = not significant. **A**–**I** *n* = 3 independent experiments. See also Supplementary Fig. 1.

Since lifetime exposure, not just smoking status, determines the risk of poor outcome in breast cancer patients [1, 2], we compared the effects of chronic nicotine to a single acute treatment in tumorsphere colony-forming assays (Fig. 1A-C). We previously showed that this assay is a robust in vitro predictor of in vivo tumor-initiation [17, 18]. In order to examine a direct effect of nicotine on breast cancer cells, we dosed with vehicle (ethanol) or various doses of nicotine every other day for two weeks before stopping treatment prior to embedding cells in methylcellulose (Fig. 1A). Since breast cancers are heterogeneous, we selected the HCC38 cell line to more closely reflect patient disease. HCC38 cells better represent the intratumoral heterogeneity found in patient cancers since they consist of both luminal and basal/stem cell types [18, 19]. We found that a 100 nM dose of chronic nicotine increased primary tumorspheres nearly 2-fold relative to vehicle-treated HCC38 cells, with no further increase at higher doses (Fig. 1B). In contrast, a single-acute treatment had little effect at any of the doses tested (Fig. 1C), consistent with the modest effects previously noted by others [9–11]. This shows that frequent exposure to low-dose nicotine is a more potent driver of tumor-initiating cell properties than even a single high dose. Self-renewal assays further showed increased secondary tumorsphere number in cells chronically treated with 100 nM nicotine relative to vehicle control (Fig. 1D, E). Since a 100 nM dose was sufficient to promote primary tumorspheres (Fig. 1B), we used this dose for all of our later experiments and refer to these as chronic nicotine (cNic) cells. Notably, this dose of nicotine is well within the normal range of serum concentrations observed after smoking [20]. The effects of chronic nicotine were not limited to HCC38 cells, as the same treatment regimen also increased primary tumorspheres nearly 2-fold in SUM149 cells (Fig. 1F). Together, our findings support a role for chronic nicotine as a more potent driver of tumor-initiating cell properties compared to acute treatment.

Nicotine serves as a ligand for the nicotinic acetylcholine receptors (nAChR), a large family of ion channels primarily expressed on neurons, but also found on a wide-array of other cell types including epithelial cells and solid tumors (15). To investigate which receptors were responsible for the effects of chronic nicotine in HCC38 cells, we examined mRNA levels for a panel of nAChR’s, including those previously shown to be expressed in breast cancer cell lines (Fig. 1G) [16]. Most abundant were the α5 and α7 nAChR’s, which we deleted with CRISPR/Cas9 (Supplementary Fig. 1A, B) to determine which receptor was required for the effects of chronic nicotine (Fig. 1B). Vector control, α5 KO and α7 KO cells were then treated with vehicle or chronic nicotine as before and assessed in tumorsphere assays. Similar to our prior results, colony formation increased roughly 2-fold in vector control cells treated with chronic nicotine (Fig. 1H, I), demonstrating the reproducibility of this effect. However, α7 KO cells were almost entirely resistant to nicotine’s effects, with little change in colony formation (Fig. 1H), while α5 KO cells still displayed enhanced colony formation (Fig. 1I). Thus, chronic nicotine directly impacts breast cancer cells through a receptor-mediated pathway that specifically requires the atypical α7, but not α5 nAChR.

### Exposure to chronic nicotine directly enhances breast cancer progression

**Fig. 2:**
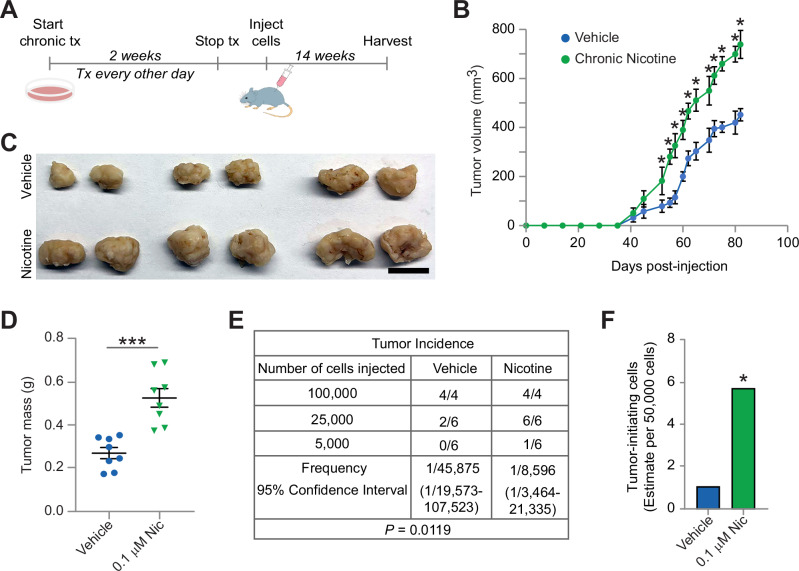
Breast cancer cell exposure to chronic nicotine drives tumor growth and initiation in vivo. **A** Schematic outlining the experimental design for HCC38 cells treated with chronic nicotine and injected into mice. **B**–**D** In vivo orthotopic breast cancer experiments comparing tumors from 500 000 HCC38 cells chronically-treated with vehicle (ethanol) or 100 nM nicotine as outlined in **A**. **B** Graph of primary tumor volume versus time after injecting cells. Statistics by two-way ANOVA and Sidak’s multiple comparisons test. **C**, **D** Primary tumors harvested after 14 weeks. **C** Representative images of vehicle or nicotine-treated tumors. Scale bar, 1 cm. **D** Final primary tumor mass for vehicle or nicotine-treated HCC38 cells. Statistics by two-tailed Student’s t-test. **B**, **D** Data represent the mean ± s.e.m. *n* = 8 tumors per cell type from 2 independent experiments. **E** Table describing the frequency of tumor formation per fat pad injected for each cell type. Results pooled from 3 independent experiments. **F** Histogram showing the estimated number of tumor-initiating cells from the data in **E**. **E**, **F** Statistics by Extreme Limiting Dilution Analysis (ELDA), which uses a chisquare likelihood ratio test to calculate *p*-values between groups. **B**, **D**, **F** *P < 0.05, ***P < 0.001.

Next, we examined the effect of chronic nicotine on breast cancer progression in vivo. In order to examine the direct effect on breast cancer cells, vehicle or cNic cells were treated prior to injecting cells into mice (Fig. 2A). Since we did not need to treat mice with nicotine, this allowed us to distinguish the cancer cell response from any local or systemic effects in the mouse. For these studies, we first orthotopically injected 500 K vehicle or cNic HCC38 cells into the inguinal mammary glands of adult female immunocompromised mice, and monitored for tumor growth. While both vehicle and cNic cells formed tumors at roughly the same time, cNic tumors grew at nearly twice the rate of vehicle controls (Fig. 2B). Similarly, we observed a final tumor mass almost 2-fold greater in cNic tumors (Fig. 2C, D). Given the increased tumorsphere colony formation and self-renewal observed in vitro (Fig. 1B), we also evaluated the tumor-initiating potential of these cells (Fig. 2E, F). For these assays, we again injected cells orthotopically into the inguinal mammary gland fat pads, only this time we compared their ability to initiate new tumors in limiting dilution assays (Fig. 2E, F). These experiments showed that cNic cells possessed a nearly 6-fold greater ability to initiate tumors relative to vehicle controls (Fig. 2E, F). Together, our mouse studies indicate that chronic treatment with low-dose nicotine directly increases both tumor growth and tumor-initiating properties in breast cancer cells. Since only the cancer cells received nicotine, these findings show that this is sufficient to robustly enhance tumor progression.

### Dynamic regulation of cell differentiation and proliferation by chronic nicotine

**Fig. 3:**
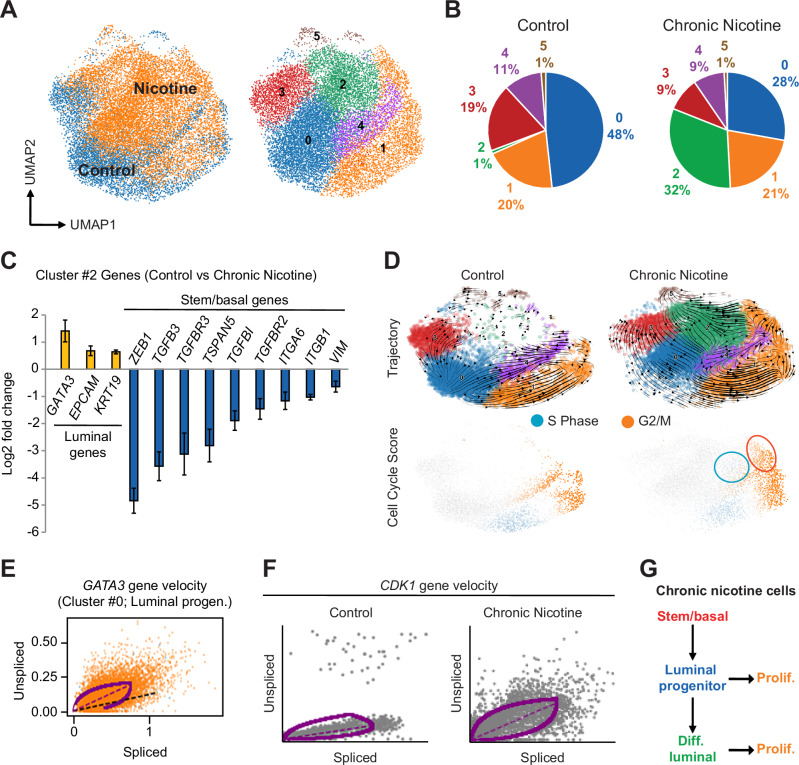
Chronic nicotine dynamically alters cell states in heterogenous disease by enhancing differentiation and proliferation. **A** Single-cell RNA-seq of HCC38 cells after chronic treatment with vehicle (6627 cells; blue) or 100 nM nicotine (9820 cells; orange). Left, Uniform manifold approximation and projection (UMAP) of all cells. Right, scanpy analysis yielded 6 discrete cell clusters at conservative resolution (0.25). **B** Pie charts depicting the frequency of each cluster in control versus chronic nicotine treated cells shown in **A**. **C** scRNA-seq data showing the relative expression of select nicotine-induced genes in cluster #2 associated with stem/basal or luminal cell types. Data represent the mean ± s.e.m. Adjusted *P* < 0.05. **D** Trajectory analysis using the dynamic model in vehicle or chronic nicotine cells. Top, UMAP and annotated clusters from each cell type are projected with their associated velocity fields displayed as streamlines. Bottom, cell cycle scores (standardized scores of mean expression levels of marker genes) for S phase (blue) and G2/M (orange). Circles highlight nicotine-induced cell populations. **E**,**F** Phase portraits for the master regulator of luminal cell fate *GATA3* **E** and the critical cell cycle checkpoint gene *CDK1* **F**. **E** Enhanced gene dynamics of *GATA3* in cluster #0 due to chronic nicotine. *GATA3* gene velocity is increased (purple line) compared to estimated steady state (black line). **F** A significant overall increase in positive velocity (purple line) of *CDK1* due to chronic nicotine. **G** Representation of the cell state changes induced by chronic nicotine. See also Supplementary Figs. 2 and 3.

The robust in vivo effects observed long after cessation of nicotine treatment (Fig. 2), suggest potential changes in cell state and gene expression programs. To perform an in-depth and unbiased assessment of how chronic nicotine impacts gene expression in different cell types, we performed single-cell RNA sequencing (scRNA-seq) in the heterogeneous HCC38 cell line. These cells consist of both stem/basal and luminal progenitor-like cell types [18, 19], allowing us to simultaneously compare differences in nicotine-induced gene expression in distinct lineages. Analysis of vehicle and cNic cells at low-resolution revealed six different clusters (Fig. 3A), including a significant increase in cluster #2 (Fig. 3B). This cluster was notable since it was essentially absent from vehicle-treated cells, but represented nearly one-third of the cNic cells (Fig. 3B). In contrast, clusters #3 and #0 were observed to decrease (Fig. 3B), while the other cell populations were unaffected. Gene set enrichment analysis (GSEA) showed enhanced expression of mammary stem/basal genes in cluster #3, and luminal progenitor markers (*KIT*, *PROM1*, *ELF5*) in cluster #0 (Supplementary Fig. 2A–C). Analysis of the key genes upregulated in cluster #2 identified markers of more differentiated luminal cell types, including *GATA3*, a master regulator of the luminal cell fate [21], *KRT19* (cytokeratin 19) and *EPCAM* (Fig. 3C). In contrast, genes associated with stem/basal cells were among the most downregulated, including *ZEB1* and *TGFBR3* (Fig. 3C). Thus, chronic nicotine increased the frequency of more differentiated luminal-like cancer cells, while simultaneously decreasing cells similar to stem/basal and luminal progenitors. These dynamic changes in cell frequency are consistent with a potential increase in differentiation.

To examine how chronic nicotine may alter the balance between different cell types, we performed dynamic trajectory analysis. This method allows us to infer changes in cell states, including potential differentiation or cell-cycle entry/exit, based on mRNA velocities [22]. Analysis of the streamlined vector fields shows increased flow from stem/basal toward luminal progenitor cells due to chronic nicotine, suggestive of differentiation (Fig. 3D). This corresponds with the decreased frequency of these cells due to chronic nicotine (Fig. 3B), and a loss of EMT or basal cell characteristics in the stem/basal population (Supplementary Fig. 3A). Indeed, we observed increased *GATA3* mRNA velocity specifically in the luminal progenitor cluster (Fig. 3E), supporting enhanced differentiation of these cells. *GATA3* is also a marker gene of cluster #2 (Fig. 3C), indicating that these cells may form due to differentiation of luminal progenitor-like cells. Additionally, we observed a characteristic eddy suggestive of proliferating cells in clusters #1 and #4 from both vehicle and chronic nicotine samples (Fig. 3D). Indeed, cell cycle gene velocities were enhanced in these two clusters, with cells entering the cell-cycle in cluster #1 and exiting in #4 (Fig. 3D and Supplementary Fig. 3B). Unexpectedly, this analysis also identified a second population of dividing cells unique to chronic nicotine that originated in cluster #2 (Fig. 3D and Supplementary Fig. 3C). These cells appear to enter S-phase in cluster #2, and then progress to G2/M in cluster #1 (Fig. 3D). The rapid proliferation of cluster #2 cells is supported by the higher velocity of the *CDK1* gene (Fig. 3F), a key indicator of cell cycle entry [23]. Thus, chronic nicotine appears to induce differentiation of a highly proliferative luminal-like cell type that may enhance overall tumor-initiating properties (Fig. 3G). Together, these discoveries highlight the ability of chronic nicotine to dynamically alter breast cancer cell states, leading to more aggressive disease.

### Innate immune gene upregulation is a conserved response to chronic nicotine

**Fig. 4:**
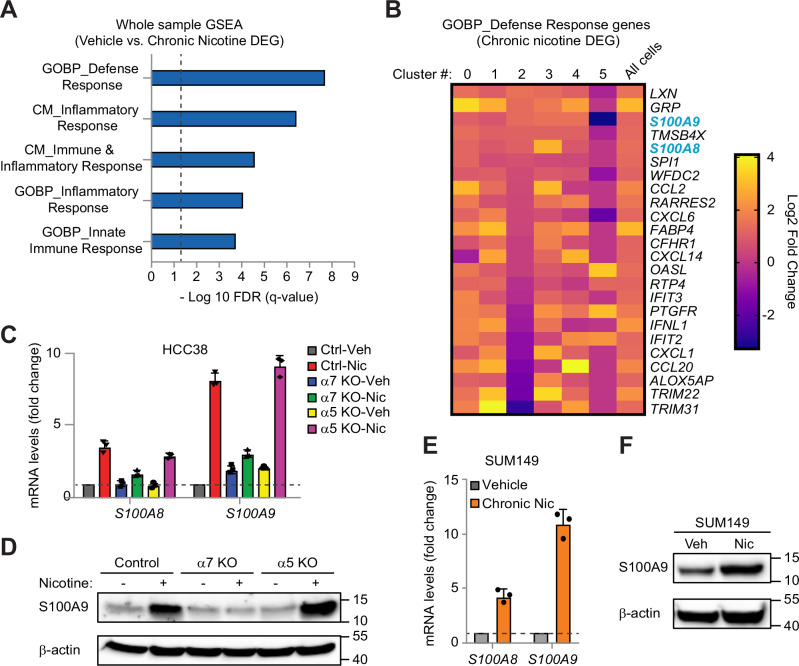
Chronic nicotine induces expression of S100A8/A9 innate immune cytokines through α7 nAChR. **A**,**B** Analysis of genes induced by chronic nicotine relative to vehicle control in all cells combined. **A** GSEA showing enrichment for innate immune gene sets due to chronic nicotine. FDR *q* < 0.05. Dashed line indicates statistical significance. **B** Heat map of the lead genes overlapping with the Gene Ontology Biological Process (GOBP) Defense Response gene set from **A** (Log2 scale). Results are also shown specific to each cluster for comparison. **C** QPCR for the indicated innate immune genes after knockout of α7 or α5 nAChR and chronic treatment with vehicle or 100 nM nicotine in HCC38 cells. **D** Representative immunoblot for S100A9 in control, α7 KO, or α5 KO cells ± chronic nicotine. **E** QPCR for *S100A8* and *S100A9* in SUM149 cells treated with chronic nicotine or vehicle control. **C**,**E** All samples were run in triplicate with GAPDH used as a loading control. For each gene, the data is normalized to the vehicle-treated vector control cells (dashed line). **F** Representative immunoblot for S100A9 in SUM149 cells ± chronic nicotine. **D**,**F** β-actin is a loading control and molecular weight markers are indicated in kilodaltons. **C**,**E** Data represent the mean ± s.e.m. *n* = 3 independent experiments. See also Supplementary Fig. 4.

Since the effects of chronic nicotine were broad, affecting multiple cell types, we investigated potential mechanisms responsible for the aggressive properties of cNic cells by performing GSEA analysis on the overall differentially-expressed genes (DEG). This includes 123 genes upregulated by chronic nicotine >2-fold. We identified significant enrichment for gene sets associated with innate immunity (Fig. 4A), suggesting possible involvement of inflammatory signaling pathways. Similar upregulation of innate immune gene sets were observed in the stem/basal and luminal progenitor clusters (Supplementary Fig. 4A, B), indicating a conserved effect in different cell types. To identify potential candidate genes responsible for the behavior of cNic cells, we compared the expression of the lead genes in the GOBP Defense Response gene set among the different clusters (Fig. 4B). This showed similar upregulation of several genes across most cell types, suggesting a common downstream response to nicotine, consistent with expression of the α7 nAChR in all clusters (Supplementary Fig. 4C). Highly represented among the conserved genes were several secreted ligands and cytokines, including: *GRP*, *S100A9*, *S100A8*, *CCL2*, *CXCL6*, *CXCL14*, *IFNL1*, *CXCL1* and *CCL20* (Fig. 4B). While breast cancer cells lack receptors for most of these, the exception was the S100A8/9 heterodimer, which binds to the widely expressed receptor for advanced glycation end products (RAGE) to enhance tumor progression [24–26]. This suggested that expression of S100A8/9 may be responsible for the effects of chronic nicotine.

To explore a role for S100A8/9 in the chronic nicotine response, we first evaluated whether α7 nAChR was required for their expression. For these studies, we examined *S100A8* and *S100A9* mRNA in our vector control, α7 KO and α5 KO cell lines treated with vehicle or chronic nicotine (Fig. 4C). Importantly, nicotine increased both *S100A8* and *S100A9* mRNA levels in vector control cells, validating our scRNA-seq results. A similar increase was also noted in α5 KO cells, however neither gene increased in the nicotine-treated α7 KO cells (Fig. 4C). Thus, α7 nAChR was specifically required for the nicotine-induced expression of *S100A8* and *S100A9*. We also observed a similar α7-dependent effect on S100A9 protein levels by immunoblot (Fig. 4D), validating our mRNA findings. This relationship was conserved in other breast cancer cell lines, as similar increases in *S100A8* and *S100A9* mRNA, as well as S100A9 protein, were also noted in SUM149 cells (Fig. 4E, F). However, these effects appear limited to TNBC cell lines, as similar chronic nicotine treatment of ER^+^ cell lines failed to significantly induce expression of either *S100A8* or *S100A9* (Supplementary Fig. 5A). To complement our gene expression findings, we also explored major signaling pathways previously shown to be activated by the α7 nAChR, such as Akt, STAT3 and ERK [27, 28] (Supplementary Fig. 5B). While all three were activated by acute nicotine at early timepoints, only phospho-STAT3 continued out to 48 hr, and persisted as long as 3 weeks post-treatment (Supplementary Fig. 5B, C), consistent with increased cytokine signaling through S100A8/A9 and RAGE [29, 30]. This chronic activation of STAT3 was also α7 nAChR-dependent, in contrast with ERK (Supplementary Fig. 5C, D). These results describe a new pathway by which chronic nicotine stimulates α7 nAChR to induce expression and activation of S100A8/A9 cytokine signaling in breast cancer cells.

### S100A8/A9 is required for the biological effects associated with chronic nicotine

**Fig. 5:**
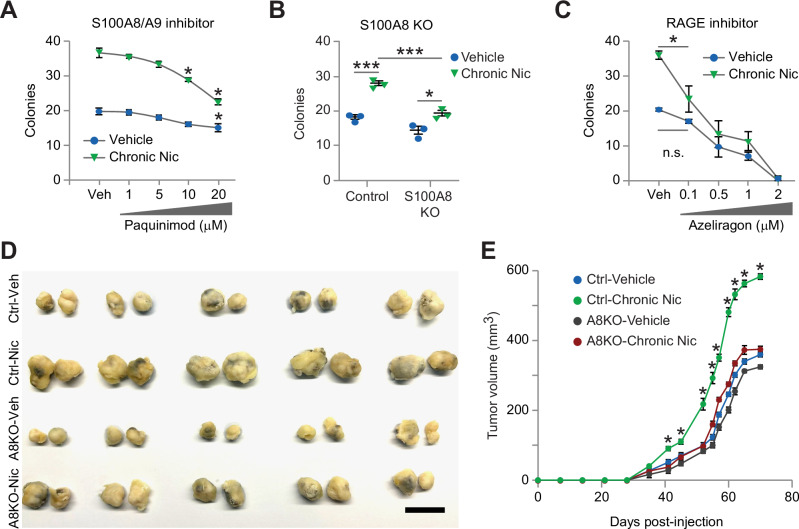
S100A8/A9 is required for the increased tumorigenicity due to chronic nicotine. **A**–**C** HCC38 tumorspheres after treatment with the indicated doses of the S100A8/A9 inhibitor paquinimod (ABR-215757) versus vehicle (DMSO) **A**, CRISPR/Cas9 knockout of S100A8 versus vector control **B**, or treatment with the RAGE inhibitor Azeliragon (TTP488) **C**. *n* = 3 independent experiments. **D** Representative images of tumors from HCC38 S100A8 KO or vector control cells treated with vehicle or chronic nicotine. Results are from orthotopic injection of 500 000 cells into inguinal mouse mammary glands and tumors harvested after 10 weeks. Scale bar, 1.5 cm. **E** Primary tumor volume versus days post-injection. **D**,**E** *n* = 16 tumors per cell type from 2 independent experiments. **A**–**C**,**E** Data represent the mean ± s.e.m. Statistics by two-way ANOVA and Tukey’s multiple comparisons test. **A**–**C** *P < 0.05 versus respective vehicle controls. n.s.=not significant. **B** *P < 0.05, ***P < 0.001. **E** *P < 0.05 for vehicle- versus nicotine-control cells and control- versus S100A8 KO-nicotine cells. See also Supplementary Fig. 5.

We next assessed the biological significance of nicotine-induced S100A8 and S100A9. For these studies, we performed methylcellulose tumorsphere assays with paquinimod (ABR-25757) (Fig. 5A), a specific inhibitor of the S100A8/A9 heterodimer that prevents binding to its receptors [31, 32]. While vehicle-treated cells showed little response to paquinimod at any of the doses tested, cNic cells displayed a dose-dependent decrease in primary tumorspheres (Fig. 5A). In fact, the maximum dose of 20 μM paquinimod reduced colony numbers to levels similar to vehicle controls. Thus, the S100A8/A9 heterodimer appears to be specifically required for nicotine-induced tumorspheres, while it was practically dispensable for colony-formation in control cells. To further investigate this role for S100A8/A9, we deleted *S100A8* with CRISPR/Cas9. Knockout of *S100A8* showed similar effects as paquinimod, reducing tumorspheres specifically in the cNic cells with little effect on vehicle controls (Fig. 5B and Supplementary Fig. 5E). We also investigated a potential role for the S100A8/A9 receptor RAGE by blocking ligand binding with the RAGE inhibitor Azeliragon (TTP488). Cells treated with chronic nicotine displayed increased sensitivity to low-dose Azeliragon (100 nM) relative to control cells (Fig. 5C), further highlighting the importance of this pathway. Finally, we examined the in vivo significance of S100A8/A9 for nicotine’s effects on tumor growth using our *S100A8* knockout cells (Fig. 5D, E). Knockout of *S100A8* also reduced mRNA levels of *S100A9* (Supplementary Fig. 5E), resulting in a functional double knockout consistent with the coordinate regulation of these two genes in other cell types [33, 34]. Again, cells were pre-treated with vehicle or chronic nicotine prior to orthotopic injection into adult female mice. The mice never received nicotine during the course of these experiments, so these studies measure nicotine’s direct impact on breast cancer cells. While chronic nicotine resulted in a nearly two-fold increase in tumor volume, similar to our prior results (Fig. 2B), *S100A8* knockout specifically blunted this nicotine-induced effect, resulting in tumors of similar size as the vehicle controls (Fig. 5D, E). Together, these genetic and pharmacological approaches support a specific role for S100A8/A9 and its receptor RAGE in mediating the effects of chronic nicotine on breast cancer cells.

### *SNPH* links S100A8/A9 signaling with smoking-related patient cancers

**Fig. 6:**
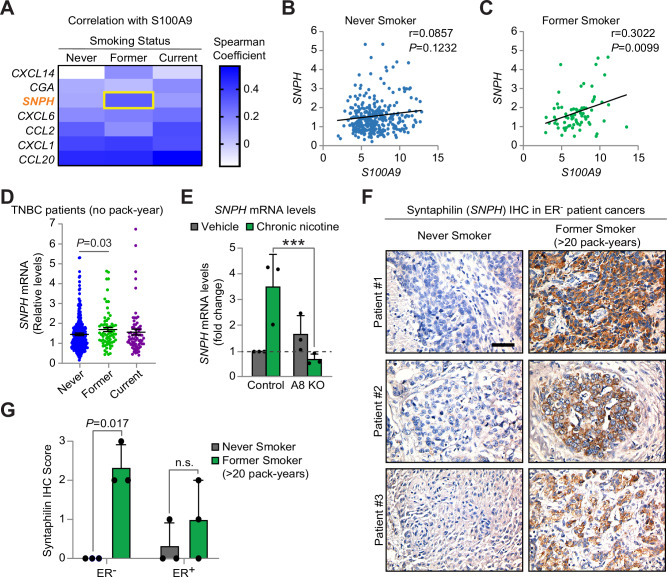
S100A8/A9 is associated with *SNPH* in smoking-related patient cancers. **A**–**D** Analysis of bulk RNA-seq data from Triple-negative breast cancer patients enrolled in the BrighTNess clinical trial, including self-reported never smokers (*n* = 325), former smokers (*n* = 72), and current smokers (*n* = 69). **A** Heat map of Spearman correlation coefficients comparing S100A9 with other nicotine-upregulated genes representing secreted signaling molecules. **B**,**C** Simple linear regression between *S100A9* and *SNPH* in never smokers **B** and former smokers **C**. **D** Expression of *SNPH* alone shows enrichment in cancers from former smokers. Lines represent the mean ± s.e.m. **A**–**D** Dots show individual patient cancers. **E** QPCR for *SNPH* in S100A8 KO or vector control HCC38 cells treated with vehicle or chronic nicotine. Samples were run in triplicate with GAPDH used as a loading control. Data is normalized to vehicle-treated vector control cells (dashed line). *n* = 3 independent experiments. **F**,**G** Syntaphilin immunohistochemistry (IHC) in patient cancers from the WHEL clinical study. **F** Images depicting Syntaphilin protein levels (brown stain) in ER^-^ breast cancers from former smokers with >20 pack-years (*n* = 3) versus never smokers (*n* = 3). Scale bar, 200 μm. **G** Syntaphilin staining intensity scores (0 = no stain, 1 = light, 2 = moderate, 3 = high) representing >25% of tumor cells in ER^-^ or ER^+^ patient breast cancers. For each subtype, *n* = 3 never smokers and *n* = 3 former smokers >20 pack-years. **E**,**G** Data represent the mean ± s.e.m. **D**,**G** Statistics by non-parametric Kruskal-Wallis ANOVA with Dunn’s multiple comparisons test.

To discern a relationship between our pathway and smoking-related patient cancers, we made use of publicly-available expression data from the BrighTNess clinical trial [35]. While the metadata included smoking status, it lacked any indication of patients’ overall lifetime exposure, such as pack-years, representing a significant limitation. Despite this drawback, we still noted a trend toward increased *S100A9* in cancers from former smokers. To probe this further, we noted that prior studies correlated S100A9 with cytokines such as CXCL1 [24]. Thus, we assessed whether other secreted signaling molecules induced by nicotine might cooperate with *S100A9* to identify smoking-related disease. This led to our discovery of *SNPH* (syntaphilin) as a gene associated with *S100A9* in former smokers, but not never smokers (Fig. 6A–C). We also noted an upward trend in current smokers. Although a few other cytokines associated with *S100A9* (CCL2, CXCL1, and CCL20), they did so independent of smoking status. In fact, *SNPH* alone enriched for cancers from former smokers (Fig. 6D), indicating it might be a potential biomarker of this disease. This finding was particularly surprising given the lack of pack-year data to stratify patients with high lifetime exposure. We further examined whether the nicotine-induced upregulation of *SNPH* may require S100A8/A9 signaling. Whereas chronic nicotine induced *SNPH* mRNA by 3.5-fold, validating our scRNA-seq findings, these levels fell to those similar to control cells in *S100A8* knockout cells (Fig. 6E). These findings highlight *SNPH* as an S100A8/A9-driven gene in breast cancer cells that may help identify smoking-related disease. Indeed, immunohistochemistry for Syntaphilin showed a specific association with ER^-^ patient samples from former smokers ( > 20 pack-years) compared to never smokers (Fig. 6F,G), consistent with our findings from the BrighTNess study and cell lines (Fig. 6D, E). In contrast, there was no clear association between Syntaphilin and ER^+^ cancers (Fig. 6G), similar to our cell line studies (Supplementary Fig. 5A). Overall, our findings highlight ER^-^ smoking-related breast cancers as a molecularly distinct subset of patient disease featuring high levels of Syntaphilin and S100A8/A9 innate immune signaling.

## Discussion

Previous clinical studies indicated that only frequent exposure to tobacco smoke results in an increased risk of breast cancer progression in patients [1, 2]. In contrast, patients with low exposure had the same risk of progression as those who never smoked [1, 2]. Thus, lifetime exposure, not just smoking status, is an important variable contributing to increased recurrence and breast cancer specific mortality. Despite this, studies designed to explore the potential mechanism responsible for this disparity have largely assessed the effects after a single high dose of nicotine [9–11]. Our study fills this gap by showing that chronic nicotine exposure significantly enhances tumor growth and initiation properties in breast cancer cells compared to acute treatment. These effects were robust and durable, lasting months after cessation of nicotine treatment due to changes in cell states, including increased differentiation, proliferation, and gene expression. This was in stark contrast to acute nicotine, where only small, transient effects were observed. Our findings highlight significant differences in nicotine’s biological activity after chronic versus acute exposure that may be responsible for the progression of smoking-related breast cancers.

To model the intratumoral heterogeneity present in patient cancers, our studies employed the HCC38 cell line. This allowed us to investigate how chronic nicotine may dynamically alter cell states in distinct breast cancer cell types with scRNA-seq studies. Our approach validated the cellular heterogeneity previously identified by surface markers [17–19], including populations of stem/basal, luminal progenitor and more differentiated cell types. Additionally, we made the unexpected discovery that chronic nicotine treatment increases differentiation toward a cell type with enhanced proliferation. These changes were associated with widespread upregulation of inflammation genes across the different cell types, including cytokines such as S100A8 and S100A9. This highlighted enhanced innate immune activation as a new property induced by nicotine signaling in breast cancer cells. Thus, by selecting HCC38’s we were able to observe dynamic changes in cell differentiation, proliferation, and gene expression due to chronic nicotine that could better reflect potential changes that occur in patient disease.

While prior studies characterized expression of nicotine receptors in breast cancer cells [16], it was unclear whether a particular receptor might be responsible for the tumor-promoting effects of nicotine. In fact, two prior studies implicated α9 nAChR as important in some breast cancer cells [9, 11]. Here, we showed that the atypical α7 nAChR is specifically required for the effects of chronic nicotine, in contrast to the more abundantly expressed α5 nAChR (Fig. 1G-I). Similar to other nicotine receptors α7 nAChR is an ion channel, however it is unique in possessing additional metabotropic signaling capability [36]. This property is linked to its critical immuno-regulatory function in multiple cell types [37–39]. Consistent with this, we observed widespread activation of innate immune genes in our cells, including cytokines such as S100A8 and S100A9. In fact, we show that α7 nAChR was required for both S100A8 and S100A9 expression, linking them for the first time. Additionally, α7 nAChR can promote differentiation in some stratified squamous epithelia [40]. Similarly, we noted increased differentiation in our chronic nicotine treated breast cancer cells, suggesting a conserved response. Together, our findings highlight a new α7 nAChR-S100A8/A9 immune signaling module that promotes breast cancer progression, and may potentially play a similar role in the response to nicotine in other cancers.

Another key discovery was that S100A8/A9 was required for the tumor-promoting effects of chronic nicotine (Figs. 4 and 5). The S100A8/A9 heterodimer is also known as calprotectin or alarmin, and is well-established for its role in breast cancer progression [24]. Previous studies focused on neutrophils as an abundant source of S100A8/A9 expression, which could signal to breast cancer cells in a feedback loop that enhanced their chemoresistance and metastasis [24]. In contrast, our data are the first to describe how nicotine can induce S100A8/A9 expression in breast cancer cells, thus acting as an alternative source of this pro-metastatic cytokine downstream of persistent cholinergic activation. Further, our approach characterized a direct effect of nicotine-induced S100A8/A9 on breast cancer cells, distinguishing it from any potential role in the tumor microenvironment (TME). Many cells within the TME can also respond to nicotine directly, as indicated by studies with fibroblasts [12] and immune cells [13]. Thus, while our findings show that chronic nicotine is sufficient to significantly enhance tumor-initiating properties in breast cancer cells, additional work is needed to define nicotine’s effects on immune and other TME cell types.

## Conclusions

Our findings detail an unexpected pro-inflammatory response induced by chronic nicotine in breast cancer cells, including secretion of S100A8/A9 cytokine, resulting in enhanced tumor progression. We further correlate this response with smoking-related patient cancers using our discovery of *SNPH* as a downstream marker of S100A8/A9 signaling. While our approach characterizes the tumor cell-intrinsic effects of S100A8/A9 on breast cancer cells, this cytokine can also affect immune cell types, suggesting the potential for additional S100A8/A9 impacts on the TME. Thus, future work is needed to delineate the tumor cell-extrinsic effects of S100A8/A9 induced by chronic nicotine on the immune response and how this may contribute to the progression of smoking related breast cancers.

## Materials and methods

### Cell lines

We purchased HCC38 cells from ATCC (Manassas, VA, USA) and SUM149 cells from BioIVT (Westbury, NY). Both cell lines were routinely tested and shown to be free of mycoplasma, and authenticated by short tandem repeat (STR) testing. HCC38 cells used in mice were additionally tested and found to be negative for an extensive panel of mouse pathogens. HCC38 cells were cultured in complete DMEM medium (DMEM supplemented with 10% fetal bovine serum (FBS) + 1% L-glutamine, sodium pyruvate, non-essential amino acids, and antibiotic/antimycotic). SUM149 cells were grown in complete Ham’s F-12 medium (Ham’s F-12 + 5% fetal bovine serum (FBS) + hydrocortisone (1 µg/mL) + HEPES (10 Mm) +insulin (5 µg/mL) + 1% antibiotic/antimycotic). Both cell lines were maintained in a humid incubator at 37 °C supplied with 5% CO2.

### Nicotine treatment

For acute treatment, we seeded 1 × 10^6^ cells into 10 cm petri-dishes before adding 0.1, 1, or 10 µM of nicotine (N3876-5ML, Sigma-Aldrich, Saint Louis, MO, USA) after 48 h. Cells were then incubated for 24 hours prior to seeding them into assays. For chronic-treated cells, we seeded 3 × 10^6^ cells before adding the same doses of nicotine every other day for 14 days, with passages performed every 3 days. For both approaches, we concurrently administered an equal volume of vehicle (ethanol) to a second dish of cells to generate appropriate controls. Vehicle and chronic-treated cells were then cryobanked at low passage for future use. For in vivo experiments, cells were treated with nicotine prior to implantation. For α7 or α5 nAChR studies, CRISPR knockout cells were generated prior to chronic dosing. In contrast, *S100A8* was deleted from cells previously treated with chronic nicotine.

### Cell transfection and lentiviral transduction for CRISPR knockout cells

Plasmids containing enhanced specificity Cas9 and the appropriate small guide RNA’s (sgRNA) in the pLentiCRISPRv2 vector were purchased from GenScript (Piscataway, NJ, USA) for generating stable knockout with lentivirus and selected using puromycin. Transient transfections for all CRISPR/Cas9 vectors into 293 T cells were performed with Lipofectamine 3000 (Invitrogen, Thermo Fisher Scientific, Waltham, MA, USA). All transfections were performed according to the manufacturers’ instructions. Stable knockout of select genes was achieved by transducing HCC38 or SUM149 cells with lentivirus produced by 293 T cells and pooling puromycin-resistant cells. A vector lacking the guide RNA was used as a negative control. Successful knockout of the respective targets was verified by Real-time QPCR.

### Single-cell RNA-sequencing

scRNA-seq libraries were prepared from HCC38 cells chronically treated with vehicle or nicotine following the 10× Genomics Single Cell 3′ V2 Reagent Kits User Guide PN-120233 (10× Genomics, Pleasanton, CA, USA). cDNA from the reverse transcription reaction was purified using DynaBeads MyOne Silane Beads (ThermoFisher Scientific) and amplified using amplification mix and primers provided in the Single Cell 3′ reagents module 1 (10× Genomics). After purification with 0.6× SPRIselect beads (Beckman Coulter, Brea CA, USA), cDNA quality and yield were evaluated using a Bioanalyzer 2100 (Agilent, Santa Clara, CA, USA). The libraries were then fragmented, end-repaired and A-tailed. After cleaning ligation products, libraries were amplified and indexed with unique sample index i7 through PCR amplification. Libraries were then submitted to the UCSD Institute for Genomic Medicine (IGM) Genomics Core for validation of library quality and sequencing on a NovaSeq 6000 (Illumina, San Diego, CA, USA).

### Single-cell RNA-seq data processing

The Cell Ranger toolkit (version 3.1.0) provided by 10× Genomics was applied to aggregate raw data, filter low-quality reads, align reads to human reference genome (GRCh38), assign cell barcodes, and generate the unique molecular identifier (UMI) matrix. A Python-based toolkit, Scanpy (version 2.3.4) [41], was used for analyzing the single-cell RNA-seq (scRNA-seq) data. Specifically, the raw UMI matrix was processed to filter out genes detected in less than 3 cells and cells with fewer than 10 genes. We further quantified the numbers of gene and UMI count for each cell, and kept high quality cells with thresholds of 600-120,000 UMIs, 400–8000 genes and less than 18% mitochondrial gene counts. Scrublet [42] was then applied to each sequencing library to remove potential doublets with the expected doublet rate of 7.6%, and cells with doubletScore larger than 90% quantile were filtered out. The normalized expression matrix was calculated based on the raw UMI counts after normalizing total counts per cell (library size) and then scaled by 1e4 and logarithmically (log1p) transformed.

### Bioinformatics analysis

Dimension reduction and unsupervised clustering were performed according to the standard workflow in Scanpy [41]. Briefly, top 8000 highly variable genes (HVGs) were detected and principal component analysis (PCA) was performed on the variable gene matrix to reduce noise, and top 50 components were used for downstream analyzes. Then, Leiden algorithm was applied at a resolution of 0.25 on such nearest neighbor graphs to detect communities and find cell clusters [43]. Differential gene expression analysis was performed with PyDESeq2, a re-implementation of DESeq2 in Python [44]. PyDESeq2 estimates variance-mean dependence in count data from sequencing assays and test for differential expression based on a model using the negative binomial distribution. The public gene expression profiles for patient triple-negative breast cancers from the BrighTNess clinical trial [35] were obtained at NCBI GEO (GSE164458).

### Trajectory analysis

Velocyto (0.6) was used to estimate the spliced and unspliced counts from the pre-aligned bam files. RNA velocity, latent time, root, and terminal states were calculated using the dynamical velocity model from scVelo (0.2.2) using standard settings. The counts matrices from CellRanger was input into the velocyto pipeline to predict cell fates. Velocyto CLI was used to estimate the spliced-unspliced count from the 10× runs. The loom files generated and pseudotime analysis performed in Python. RNA velocity latent time, root, and terminal states were calculated using the scVelo package implementation in Scanpy 1.9.1. The scv.pp.filter_and_normalize function was used to filter, normalize and preprocess the annotated data. The gene-specific velocities were computed from the spliced and unspliced abundances using scv.tl.velocity (mode = ‘dynamic’) and scv.tl.velocity_graph functions. These velocities were visualized over the precomputed UMAP embedding using scv.pl.velocity_graph or scv.pl.velocity_embedding_grid functions.

### Real-time qPCR

qPCR experiments on cultured cells were performed by collecting total RNA using the RNeasy Mini Kit (Qiagen) and reverse transcribing with the High-Capacity cDNA Reverse Transcription Kit (Applied Biosystems, Thermo Fisher Scientific) according to manufacturer instructions. Real-time qPCR was performed using SYBR™ Green PCR Master Mix (ThermoFisher Scientific, Waltham, MA, USA) and run on a LightCycler 480 qPCR System (Roche, Basel, Switzerland). See Supplementary Table 1 for a list of primers.

### Immunoblotting

Whole cell lysates were prepared from cell lines with RIPA lysis buffer (100 mM Tris pH 7.5, 150 mM sodium chloride, 0.1% deoxycholate, 0.1% SDS, 50 mM NaF, Protease inhibitor cocktail (Roche), 2 mM PMSF, 2 mM sodium orthovanadate) combined with scraping and the lysates cleared by centrifugation. Protein concentrations were quantified using a Pierce™ BCA Protein Assay Kit (ThermoFisher Scientific). Standard Western blotting procedures were performed. Primary antibodies were used for immunoblotting at the following dilutions: 1:1000 S100A9 (Cat# 72590), p-AKT (Cat# 8599), total Akt (Cat# 3063), p-STAT3 (Cat# 9131), total STAT3 (Cat# 9139), p-ERK(1/2) (Cat# 4370), total ERK(1/2) (Cat# 4696), and 1:2000 HSP90 (Cat# 4874), all from Cell Signaling Technology, Danvers, MA, USA, as well as 1:2000 β-actin (Cat# MABT825, MilliporeSigma, Burlington, MA, USA). Imaging was performed with a c300 Chemiluminescent Western Blot Imager (Azure Biosystems, Dublin, CA, USA). All blots or gels derive from the same experiment and were processed in parallel. See Supplementary Information for unedited blots (Supplementary Fig. 6A, B).

### Immunohistochemistry

Immunohistochemical staining of formalin-fixed paraffin-embedded (FFPE) breast cancer sections was performed with de-identified patient tissues from the WHEL clinical study [45], purchased from the Moores Cancer Center biorepository. Antigen retrieval was performed in citrate buffer at pH 6.0 and 95 °C for 20 min before blocking in 5% normal goat serum diluted in Tris-buffered saline, pH 7.6/ 0.25% Tween-20 (TBST). Sections were then incubated in Syntaphilin primary antibody at 1:300 dilution (Cat# HPA049393; MilliporeSigma, Burlington, MA, USA) overnight at 4 °C followed by biotin-conjugated anti-rabbit IgG and an avidin–biotin peroxidase detection system with 3,3’-diaminobenzidine (DAB) substrate, and counterstained with hematoxylin. All slides were imaged on a Nikon Ti/E inverted microscope using Nikon Elements software. Stained tumor sections were scored by a blinded observer.

### Tumorsphere assays

Primary tumorsphere formation was assessed in cells grown under anchorage-independent conditions in methylcellulose. HCC38 (5000) or SUM149 cells (10,000) were cultured in 0.9 mL of 1% methylcellulose/complete DMEM medium in ultra-low adhesion 24-well dishes (Corning, Corning, NY, USA) and cells cultured for 12–14 days. Primary tumorspheres were assessed by a blinded observer by counting colonies consisting of at least 6 cells from 4 fields per well with a 10x objective. Blinding was performed by having one individual set-up the experiment, coding each well, and a different individual count the colonies. We measured self-renewal by collecting primary tumorspheres by dilution in at least 3 volumes of PBS, dissociating them with trypsin for approximately 10 min, and re-seeding in 1% methylcellulose before evaluating secondary colonies after 10 days. For experiments with the S100A8/A9 inhibitor paquinimod (ABR-25757) or the RAGE inhibitor Azeliragon (TTP488) (Selleckchem, Houston, TX, USA), a single dose was added only once when embedding cells, and compared against cells receiving the same volume of vehicle (DMSO).

### Orthotopic breast cancer

Tumors were generated by injection of HCC38 cells diluted in 50 μl sterile PBS into the inguinal fat pads of 8- to 10-week-old adult female nonobese diabetic/ severe combined immunodeficiency/ interleukin-2 receptor γ chain knockout (NSG) mice (purchased from UCSD Animal Care Program colony). Mice were randomized by body weight prior to tumor cell injection. All mice were monitored weekly for tumor formation by gentle palpation. Most tumors formed within 8–10 weeks. Tumor volume was measured with calipers twice weekly by a blinded observer. Blinding was readily achieved by having one individual perform the injections, referring to each mouse only by code, and another different individual responsible for detecting and measuring the tumors. The experiment was concluded, and mice were sacrificed just prior to the tumors reaching the maximum allowable size of 2 cm^3^ or 14 weeks. Estimated tumor initiating cell frequencies were calculated with the Extreme Limiting Dilution (ELDA) web-based tool [46]. Primary tumor mass was determined by assessing the wet weight of the resected tumors. Remaining tumor-free mice were harvested at 14 weeks and the absence of any detectable tumor was confirmed at necropsy. Sample size was selected based on prior experiments with the specific number of mice per condition indicated in the figure legends.

### Ethics

All mouse studies described were approved by the UCSD Institutional Animal Care and Use Committee (IACUC) and performed in accordance with the guidelines set forth in the NIH’s Guide for the Care and Use of Laboratory Animals (National Academies Press, 2011).

### Statistical analysis

Data presentation and statistical tests are indicated in the figure legends. Two-tailed Student’s t-tests were used for comparing two means while ANOVA was performed for 3 or more data sets. Post-hoc analysis was performed using an appropriate multiple comparison test as indicated in the legends. For all analyzes, *P* < 0.05 was considered statistically significant. Statistical analysis was performed using GraphPad Prism software (San Diego, CA, USA).

## Supplementary information

Supplementary Information

## Data Availability

RNA sequencing data that support the findings of this study have been deposited in the NCBI Sequence Read Archive (SRA) with the primary accession code PRJNA1145480.
