# Supplementary material for: S100A8/A9 innate immune signaling as a distinct mechanism driving progression of smoking-related breast cancers
Source: Oncogene. 2025 Jan 25;44(15):1051–62. doi: 10.1038/s41388-025-03276-5 (PMC11976259; doi:10.1038/s41388-025-03276-5)
Supplement: Supplementary file 1 — Supplementary Information [file 41388_2025_3276_MOESM1_ESM.pdf]

Supplementary Figure 1

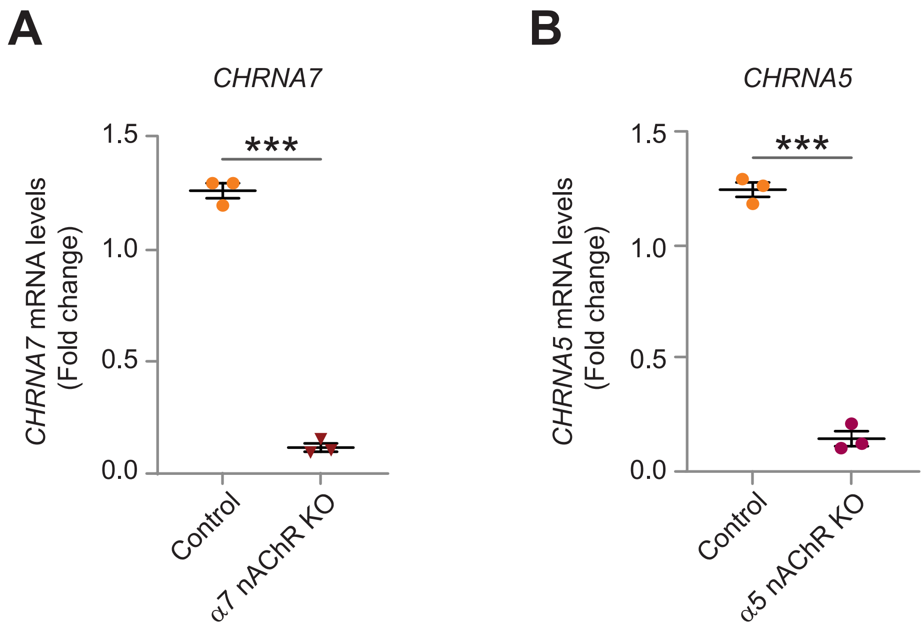

Supplementary Figure 1. Related to Figure 1. (**A,B**) Validation of reduced mRNA expression after CRISPR/Cas9 gene excision of the α7 (**A**) or α5 (**B**) nicotinic acetylcholine receptors (nAChR) in HCC38 breast cancer cells. Data from QPCR experiments showing the levels of *CHRNA7* (**A**) or *CHRNA5* mRNA (**B**) in the respective vector control or knockout cells relative to parental cells. Statistics by student's t-test. ****P*<0.001. All samples were run in duplicate with GAPDH as a loading control. Data represent the mean ± s.e.m. n=3 independent experiments.

Supplementary Figure 2

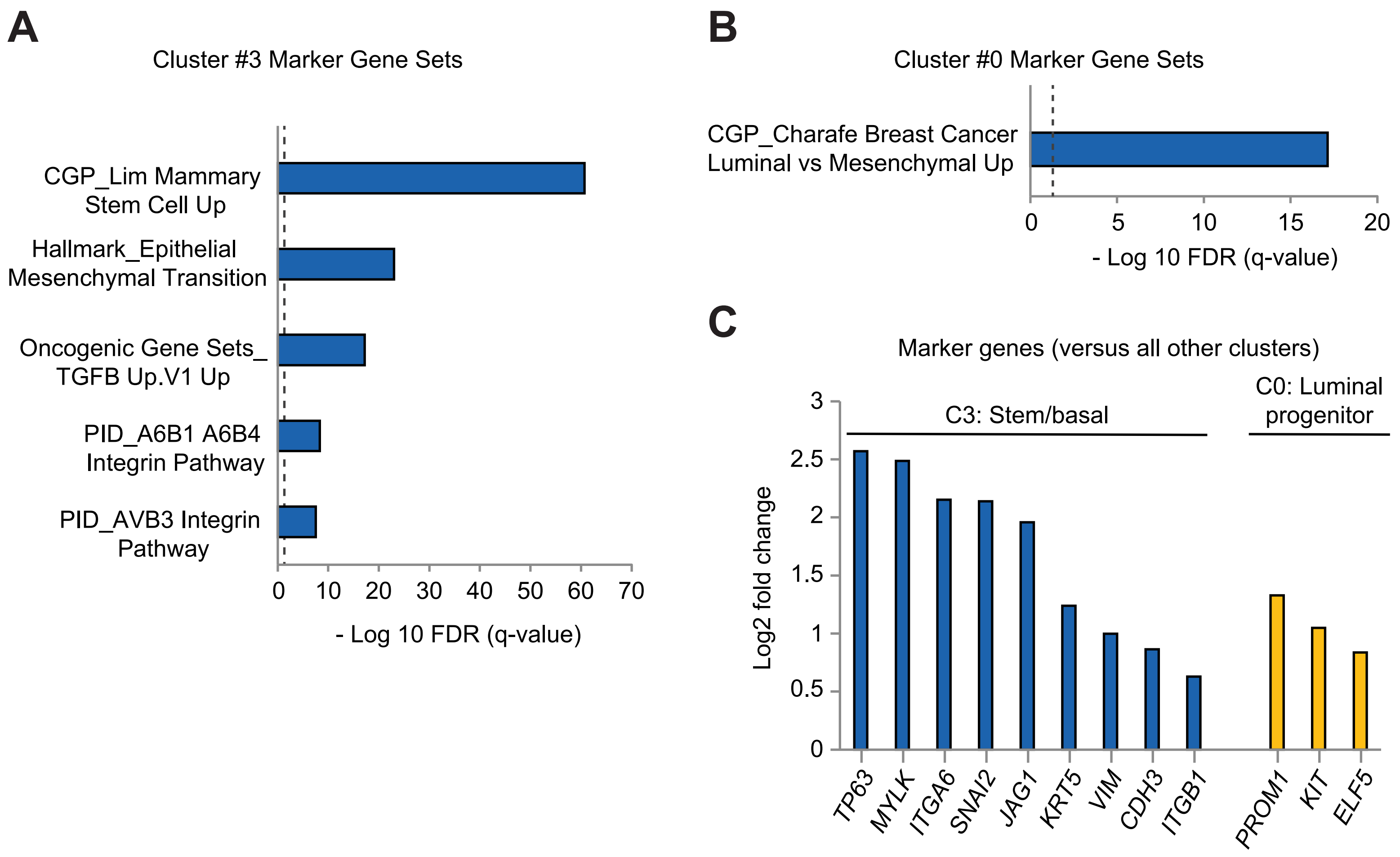

Supplementary Figure 2. Related to Figure 3. (**A-C**) Analysis of clusters #3 and #0 marker genes from scRNA-seq data. (**A,B**) Gene set enrichment analysis (GSEA) for marker genes specifically associated with cluster #s 3 (**A**), and 0 (**B**). (**C**) Histogram displaying the relative mRNA expression levels of select stem/basal and luminal progenitor cell marker genes in clusters #3 and #0 respectively. Bars represent the log2 fold change compared to cells in all other clusters. FDR $q < 0.05$. Dashed lines indicate statistical significance.

Supplementary Figure 3

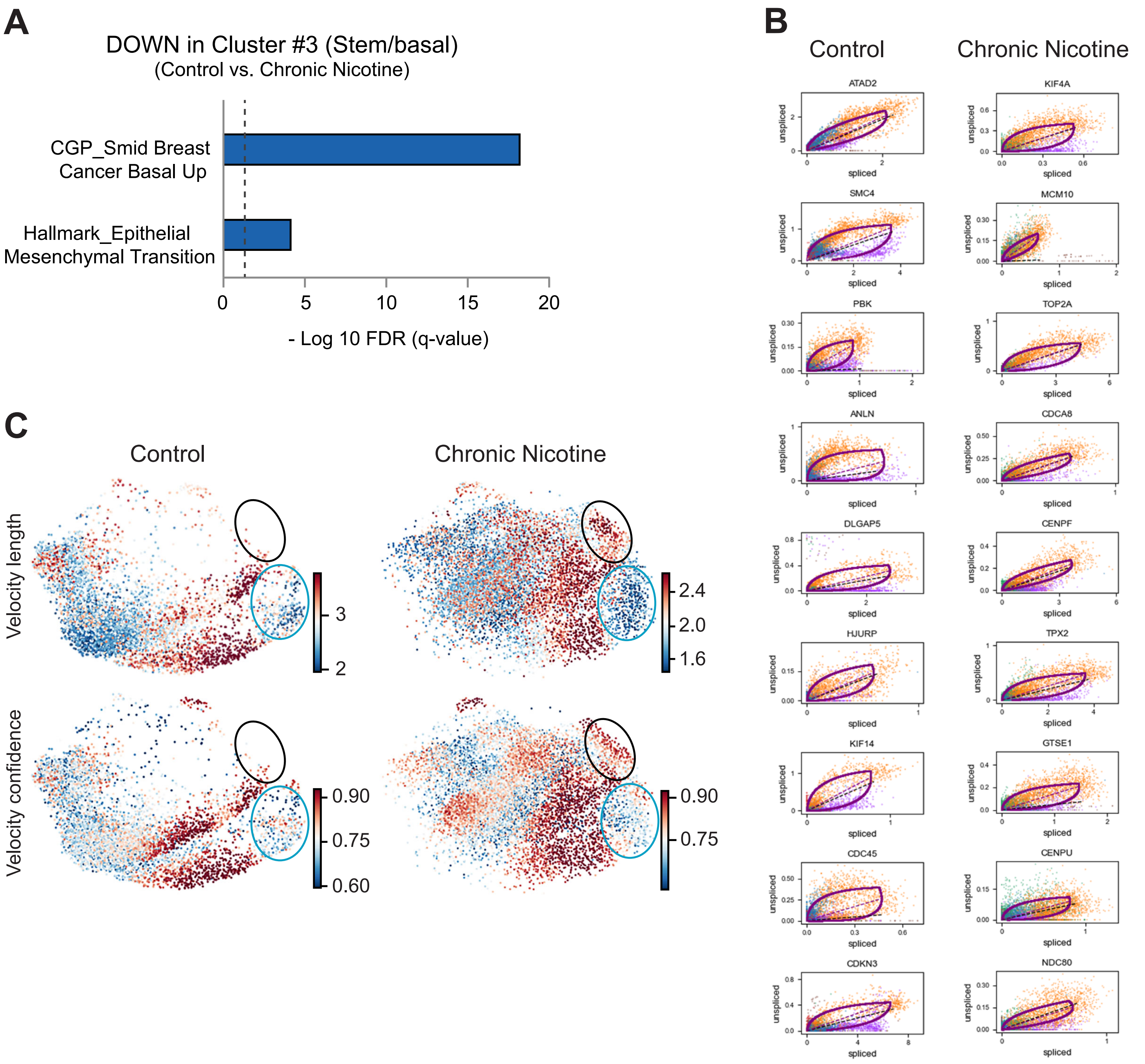

Supplementary Figure 3. Related to Figure 3. (**A**) GSEA for genes down in the stem/basal cluster #3 due to chronic nicotine. FDR q < 0.05. Dashed lines indicate statistical significance. (**B**) Phase portraits for the top dynamic genes in cluster #1 (orange), reveal numerous cell cycle-related genes, many of which are turned off as they progress to cluster #4 (purple). (**C**) Dynamic trajectory modeling data analyzed for speed and coherence. UMAP data projected with their associated velocity length and confidence fields shows an increased rate of cell cycle entry in the nicotine-induced cluster #2 cells (black ovals) relative to proliferating cells in the control (blue ovals).

Supplementary Figure 4

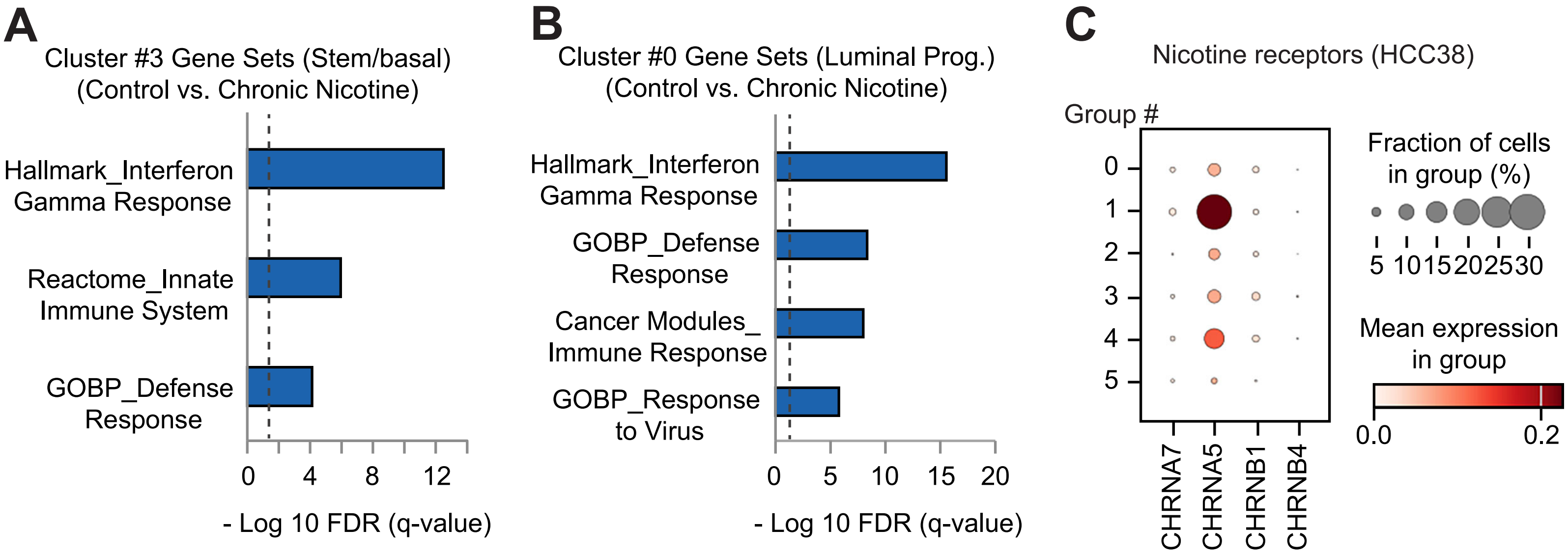

Supplementary Figure 4. Related to Figure 4. (**A,B**) GSEA for genes upregulated due to chronic nicotine in clusters #3 (**A**) and #0 (**B**). FDR q < 0.05. Dashed lines indicate statistical significance. (**C**) Dot plot displaying the relative mRNA expression levels and frequency of select nicotinic acetylcholine receptor genes in the different cell clusters identified by scRNA-seq.

Supplementary Figure 5

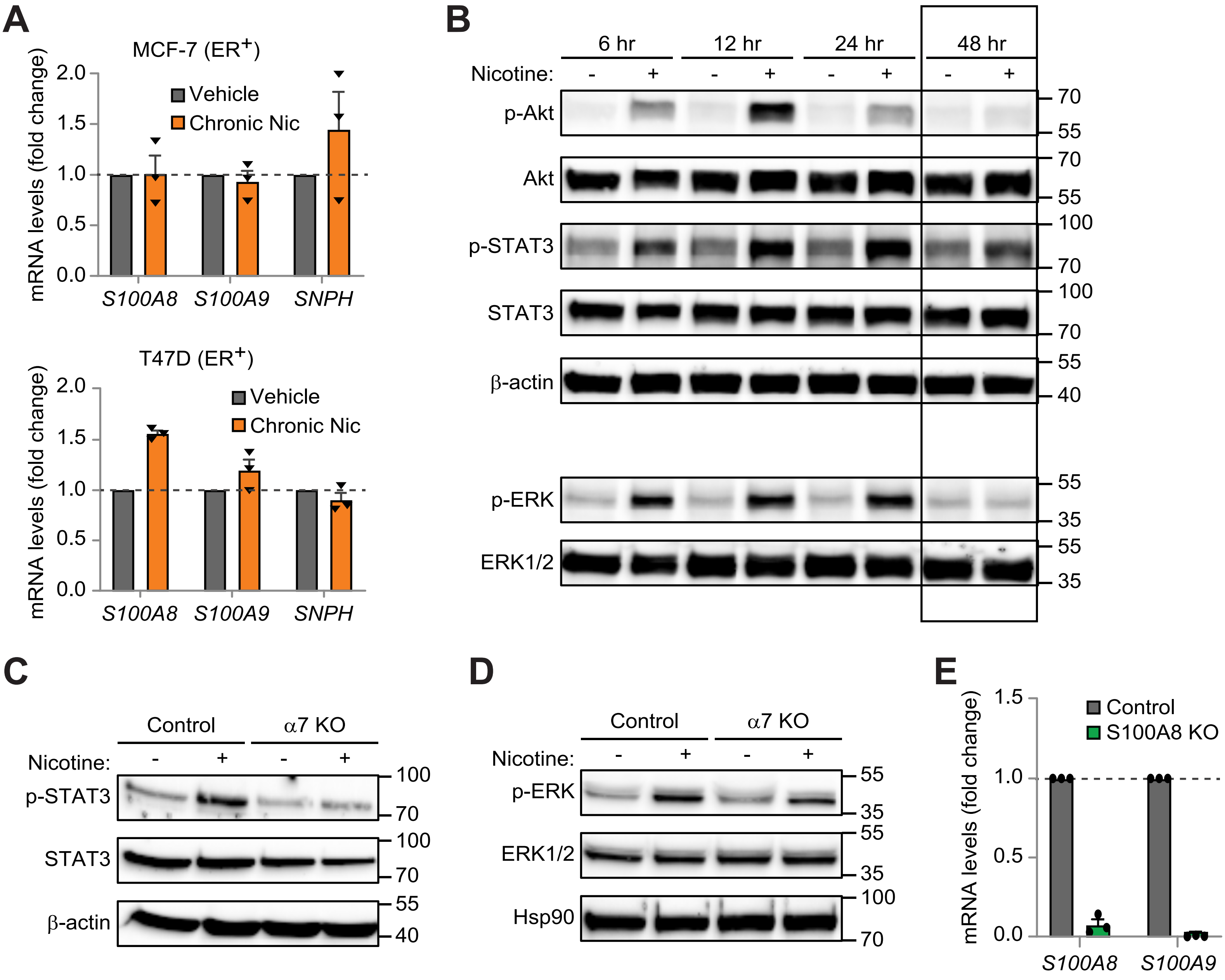

Supplementary Figure 5. Related to Figures 4 and 5. (**A**) QPCR for the indicated genes after chronic treatment with vehicle or 100 nM nicotine in ER$^+$ MCF-7 and T47D breast cancer cell lines. For each gene, the data is normalized to the vehicle-treated control cells (dashed line). n=3 independent experiments for each cell line. (**B**) Immunoblot timecourse showing activation of major signaling pathways at the indicated timepoints after treatment with vehicle or 100 nM nicotine. A box around the 48 hr timepoint emphasizes reduced signaling of all but phospho-STAT3. The space indicates that the blots for phospho- and total ERK were performed with the same samples run on a separate gel. (**C,D**) Representative immunoblots from control versus α7 KO HCC38 cells +/- nicotine. (**C**) Chronic nicotine-treated cells harvested 3 weeks post-treatment. (**D**) Acutely treated cells with vehicle or 100 nM nicotine were harvested after 12 hours. (**C,D**) β-actin or Hsp90 are loading controls and molecular weight markers are indicated in kilodaltons. n=3 independent experiments. (**E**) QPCR experiments showing reduced *S100A8* and *S100A9* mRNA expression after CRISPR/Cas9 gene deletion of *S100A8* in HCC38 breast cancer cells. For each gene, data is relative to vector control cells (dashed line). n=3 independent experiments. (**A,E**) Samples were run in triplicate with GAPDH used as a loading control. Data represent the mean ± s.e.m.

Supplementary Figure 6

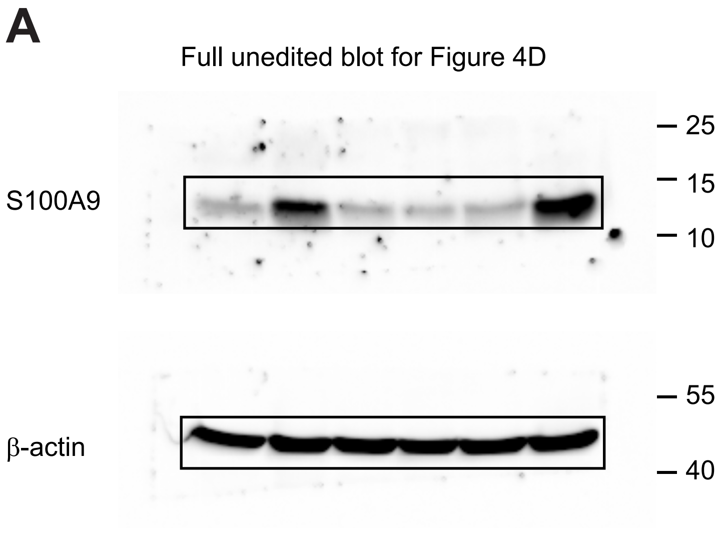

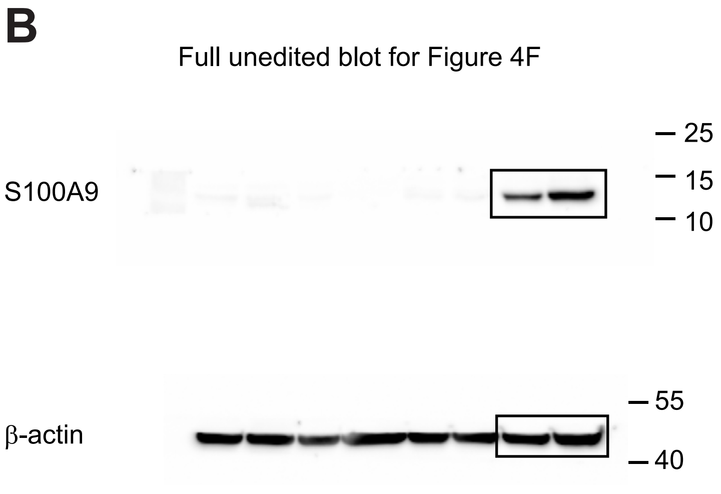

Supplementary Figure 6. Unedited Western blots. (**A,B**) Unprocessed scans for the immunoblot data shown in main figures 4D (**A**) and 4F (**B**). Boxes outline the cropped areas used in figures. Molecular weight markers are indicated in kilodaltons.

Supplementary Table 1. Primers for QPCR

Primers	Forward (5´ to 3´)	Reverse (3´ to 5´)
CHRNA1	CTGGACCTACGACGGCTCT	CGCTGCATGACGAAGTGGT
CHRNA2	AGGCTCGCATACCGAGACT	TCACCACGTCTGAAGTGTTGG
CHRNA3	TGAGCACCGTCTATTTGAGCG	TGGACACCTCGAAATGGATGAT
CHRNA4	GGAGGGCGTCCAGTACATTG	GAAGATGCGGTCGATGACCA
CHRNA5	ACGTTTTGAAGGGACCAGTACG	ACTCACAATCTCCCATTCTCCAT
CHRNA6	GGCAGGGATTCCTTCATGGG	GCCTCTCCTCAGTTGCACAG
CHRNA7	GCTGGTCAAGAACTACAATCCC	CTCATCCACGTCCATGATCTG
CHRNA9	CAGAGACGGCAGATGGAAAAT	CCACTGGACGAAGAGCATTAGAA
CHRNA10	TCGACATGGATGAACGGAACC	ATCGTAGGTAGGCATCTGTCC
CHRNB2	GGTGACAGTACAGCTTATGGTG	AGGCGATAATCTTCCCACTCC
CHRNB3	TGCTGGTTCTCATCGTCCTTG	GCATCTTCATTTTCGGCGATTGA
CHRNB4	CAGCTTATCAGCGTGAATGAGC	GTCAGGCGGTAATCAGTCCAT
CHRNG	ACGAGACTCGGATGTGGTCAA	GACACCGTCCACGTTGTTCT
CHRNE	GTGGATGCCGTGAACTTCGT	GCACCCAGTCGGACACTTC
CHRND	GGAGAACAACAATGACGGCTC	TGGCAGCCAGTACACGAAG
S100A8	ATGCCGTCTACAGGGATGAC	ACTGAGGACACTCGGTCTCTA
S100A9	GGTCATAGAACACATCATGGAGG	GGCCTGGCTTATGGTGGTG
SNPH	CCAGGAAGTAGACGGACCTCT	CTGCCCTTGTAGGAGCCAG
